# Variance due to the examination conditions and factors associated with success in objective structured clinical examinations (OSCEs): first experiences at Paris-Saclay medical school

**DOI:** 10.1186/s12909-024-05688-5

**Published:** 2024-07-02

**Authors:** Coralie Amadou, Raphael Veil, Antonia Blanié, Claire Nicaise, Alexandra Rouquette, Vincent Gajdos

**Affiliations:** 1 Paris-Saclay Medical School, Kremlin-Bicêtre, France; 2 Department of Endocrinology and Diabetology, Hôpital Sud-Francilien, Corbeil-Essones, France; 3 Department of Public Health and Epidemiology, Hôpital Bicêtre, Assistance Publique Hôpitaux de Paris, Le Kremlin Bicêtre, France; 4 Paris-Saclay University, UVSQ, Inserm, CESP, Paris, France; 5 General Pediatrics Department, Hôpital Antoine Beclère, Assistance Publique Hôpitaux de Paris, Clamart, France; 6 Département d’Anesthésie-Réanimation-Médecine PériOpératoire, Hôpital Bicêtre, Assistance Publique Hôpitaux de Paris, Le Kremlin Bicêtre, France; 7 Department of Diabetes and Endocrinology, Sud- Francilien Hospital, Paris-Saclay University, Corbeil-Essonnes, France

**Keywords:** Medical education, Objective structured clinical examinations, Multilevel analysis

## Abstract

**Background:**

We aimed to measure the variance due to examination conditions during the first sessions of objective structured clinical examinations (OSCEs) performed at a French medical school and identify factors associated with student success.

**Methods:**

We conducted a retrospective, observational study using data from the first three OSCEs sessions performed at Paris-Saclay medical school in 2021 and 2022. For all sessions (each organized in 5 parallel circuits), we tested a circuit effect using a linear mixed-effects model adjusted for sex and the average academic level of students (according to written tests). Then, we studied the factors associated with student success at one station using a multivariate linear mixed-effects model, including the characteristics of students, assessors, and standardized patients.

**Results:**

The study included three OSCEs sessions, with 122, 175, and 197 students and a mean (± SD) session score of 13.7(± 1.5)/20, 12.7(± 1.7)/20 and 12.7(± 1.9)/20, respectively. The percentage of variance due to the circuit was 6.5%, 18.2% (statistically significant), and 3.8%, respectively. For all sessions, the student’s average level and station scenario were significantly associated with the score obtained in a station. Still, specific characteristics of assessors or standardized patients were only associated with the student’s score in April 2021 (first session).

**Conclusion:**

The percentage of the variance of students’ performance due to the examination conditions was significant in one out of three of the first OSCE sessions performed at Paris-Saclay medical school. This result seems more related to individual behaviors rather than specific characteristics of assessors or standardized patients, highlighting the need to continue training teaching teams.

**National clinical trial number:**

Not applicable.

**Supplementary Information:**

The online version contains supplementary material available at 10.1186/s12909-024-05688-5.

## Introduction

Objective Structured Clinical Examinations (OSCEs) assess medical students’ knowledge and clinical skills in a simulated environment. In addition, they are of great pedagogical interest. OSCEs have been widely used for several decades in several countries, such as Canada and the USA [1, 2]. However, the validity of OSCEs depends on their rigorous implementation in accordance with Messick’s concept [3]. And validity becomes even more important when the consequences of OSCEs can have a long-term impact on the students’ curriculum, which then requires more evidence of the interpretative validity of the results (Kane’s validity framework) [4].

In France, OSCEs are progressively being integrated into the assessment of medical students, and they will soon be mandatory in the framework of an ongoing national reform of medical education. Indeed, starting in 2024, national OSCEs (that will involve approximatively 10,000 medical students per year) will account for 30% of the 6th -year student’s overall score for the choice of specialty and place of post-medical school training (equivalent to residency in the USA). Therefore, OSCEs will play a determinant role for French medical students, which raises legitimate concerns from French medical teachers [5]. In this context, the Paris-Saclay medical school (Paris-Saclay university, France) introduced the first OSCEs into its curriculum in 2021.

To assess the students fairly and efficaciously with respect to the knowledge and skills expected within the OSCEs, it is essential to minimize the inter-circuit heterogeneity by rigorous standardization of the evaluation conditions (including scoring by assessors, standardized patients’ behavior, premises, and equipment). Notably, this requires the assessors and standardized patients to receive training and practice to fill the standardized scoring scale.

After these first experiences, it appeared necessary to assess the quality of these OSCEs sessions. Several metrics have been proposed by the Association for Medical Education in Europe (AMEE) [6]. One of them is the between-group (i.e., between-circuit) variance assessment. In its guidelines, the AMEE stated that this between-group variance should be under 30%. Indeed, the principle is that all the variance in student scores should result from the differences in student performance, not from differences in examination conditions. Therefore, the first objective of our study was to compute the percentage of variance due to the examination conditions (i.e., the circuit). This metric indicates the uniformity of the assessment process between groups. The second objective of this retrospective analysis was to identify associations between students, assessors, and standardized patients’ characteristics and students’ station score.

## Methods

We performed a retrospective, observational study using data from the three first OSCEs sessions organized at the Paris-Saclay medical school. The Paris-Saclay medical school organized its first OSCE (non-mandatory and mock) session in April 2021 for 4th year medical students, followed by a mandatory, genuine evaluation session in December 2021 for 5th year medical students. Therefore, participants of December 2021 corresponds, for the most part, to the students of April 2021 who had advanced to the next academic year. A third session, also counting for assessment, took place in April 2022, for 4th year medical students. During these sessions, students were randomly allocated to one of 5 parallel circuits, each containing five stations. A medical scenario was set up for each station with identical scenarios for all five circuits. The clinical scenarios were developed and validated by a pedagogical and cross-disciplinary committee. For each, one teacher (specialist of the discipline) wrote the first draft and then it was reviewed by the committee until a final version was approved by every member of the committee.

### Organization of the OSCEs, recruitment, and training of participants

**Fig. 1:**
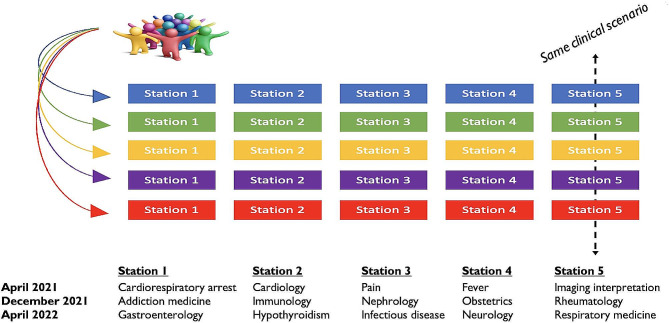
OSCEs organization and station themes (clinical scenarios)

The Fig. 1 shows the organisation of the three OSCEs sessions. Each circuit is designated by a color name, and each circuit comprised five stations with specific clinical scenarios (numbered from 1 to 5, the number designating the clinical situation evaluated and not the order of passage) which were the same between parallel circuits.

Each station was manned by the assessor in charge of scoring the student using a standardized scoring scale, and a standardized patient (or “facilitator” in the absence of simulation requirement). The medical school non-randomly selected the assessors among the hospital and teaching personnel. To ensure that the largest number of the medical school teachers experience at least one session of OSCEs, the medical school tried to select, for each session, teachers not involved in the previous one. Junior and middle-grade physicians were required to participate, while senior physicians (professors) could participate on a voluntary basis. The assessors were required not to be experts in the medical specialty being evaluated in their station.

For these first OSCEs sessions, the medical school non-randomly selected standardized patients among amateur actors, students with experience in theatre performance, and hospital and teaching staff.

The April and December 2021 sessions were scheduled on a single half-day. Each required 25 fixed pairs of participants (one assessor and one standardized patient for all five stations of all five circuits). The session of April 2022 was organized to take place on a full day. Due to the lunch break for participants in a station, the medical school constituted a larger pool with 30 non-fixed pairs (but allocated in the same station and circuit for the day). To summarize, the variance in the results due to the participants in a station could not be distinguished between assessors and standardized patients for April and December 2021 as the pairs were fixed. In contrast, for the third session, this variance could be distinguished as the pairs were not fixed during the examination day.

All participants in a station attended a first and general preparatory meeting prior to the first OSCEs session, and a second specific training session for their station, where they were apprised of the clinical scenario. The authors of the clinical scenario conducted these specific meetings. The standardized scoring scales (including the specific items of knowledge and skills being evaluated) were tested, as were the electronic tablets that were to be used during the OSCEs, so that the assessors could familiarize themselves with the material. The standardized patients learned their role in the OSCEs and were trained to play out the scenario using the equipment if needed.

The students were randomly allocated to the circuits. Using a list of the names of all students, groups of equal size were formed to create the circuits, and then sub-groups were defined to schedule the passage times for each station.

### Data collection

For each student, we collected the score obtained on each station of an OSCEs session, as well as the overall score for the session. We also collected, based on written tests (multiple-choice questionnaires), the semester-level average academic scores of students (not including the OSCEs) during their medical studies. For students in 4th year of medicine, the controlling average score for each student was calculated as the average over both the first and second semesters of the academic year. For students in 5th year of medicine, the controlling average score for each student was calculated as the average of the scores from the 2 semesters of their 4th year and the scores from the 1st semester of 5th year as the OSCEs occurred during the 5th year second semester.

We identified all the participants in a station (encompassing assessors and standardized patients) from the OSCEs schedules provided by the medical school. A questionnaire was sent to all of them. They were asked to confirm that they had participated as planned in the OSCEs. They also provided additional information, including age, sex, job position, medical specialty (if physicians), and the number of years of professional experience (if physicians). For those who did not respond to the questionnaire, the requested data were obtained from the medical school, publicly published decrees (for specific nominations), or other official medical and institutional sources.

Based on the data thus obtained, assessors were categorized as: junior doctors, middle-grade doctors (hospital practitioners with or without university teaching functions), assistant/associate professors (equivalent to university lecturers), and professors. Standardized patients were classified into two categories for the purposes of statistical analysis, as follows: non-physicians, residents, or junior doctors; vs. senior doctors (including middle-grade doctors and above). The choice of only two categories was made because of an unbalanced distribution of the profiles of standardized patients among stations that did not allow sufficient variability to distinguish the association between the obtained scores and specific profiles.

Physicians were classified as belonging to one of 5 specialties: general (family) medicine (including public health doctors), medical specialties (including pediatrics), surgical specialties, paraclinical specialties (medical imaging, biology, pathology), anesthesiology and critical care.

### Statistical analysis

#### Description of data

The characteristics of the participants in a station are described as number and percentage for qualitative variables, and mean ± standard deviation (SD) for quantitative variables. The distribution of the student’s scores on each OSCEs session is represented using box plots, for each circuit and for each session. The relationship between the total score obtained on one OSCEs session and the average score during the second cycle of medical studies was studied using scatterplots with linear regression fitting, firstly overall, and secondly, distinguishing each circuit, for each session.

#### Multilevel analysis (variance due to the circuit)

For each session, the percentage of variance due to the circuit was computed using a linear mixed effects model, with an adjustment for the average academic score of students (as described above), and the student’s sex. The existence of a random effect related to the circuit was tested by calculating the likelihood ratio between the different models (with and without the random effect).

#### Factors associated with success in a station

We performed a multivariate analysis to study the association between the score obtained by a student in a station (outcome) and the characteristics of students (age, average academic score), assessors (sex, years of experience, job position, medical specialty), standardized patients (sex, years of experience, job position, and medical specialty if applicable), and the topic being examined in the station (clinical scenario). These associations were studied as fixed effects. We also included random effects regarding students, pairs of participants in a station (April and December 2021), and assessors and standardized patients separately (April 2022) in the multivariate model.

All analyses were performed using R version 4.0.1 (2020-06-06). Graphs were plotted with the “ggplot2” package and mixed models were computed with the “lme4” package. All tests were two-sided and a *p*-value < 0.05 was considered statistically significant.

#### Ethics statement

All participants in the study (students, assessors, and standardized patients) were given information about their right to refuse the use of their data for research purposes. Informed consent was obtained from all subjects, in compliance with the French General Rules for Data Protection. All data were made anonymous for analysis. The study was validated and registered by the data protection department of the University of Paris-Saclay. The approval of an ethics committee was not required as this study was exclusively based on data analysis and not involving the Human person according to the French Public Health Code (Loi Jardé—n°2012–300 of March the 5th 2012, in application in November 2016—Article R1121-1).

All methods were carried out in accordance with relevant guidelines and regulations (STROBE guidelines for reporting observational research and the French General Rules for Data Protection for the management of personal data).

## Results

### Study population and exam sessions

The three OSCEs sessions examined 122, 175 and 197 students in April 2021, December 2021 and April 2022 respectively, of whom 71%, 69% and 66% respectively were women. A score was available for every student in each station, for an overall total of 610, 875 and 985 scores respectively.

The characteristics of the participants in a station (assessor and standardized patient) are shown in Table 1. Data relating to the position and specialty (for physicians) were available for 100% of doctors. The exact number of years of professional experience since graduation (end of the third cycle of medical studies) was available for 50% of assessors and 21% of standardized patients (when post-graduate physicians). In view of the high rate of missing data for this variable, it was not included in the multivariate analysis.

**Table 1:** Characteristics of the participants in a station (assessors and simulating patients)

Session	April 2021		December 2021		April 2022	
	25		25		30	
**Assessors (** ***n*** **)**						
Position, *n* (%)						
Junior doctor	17	(68.0)	20	(80.0)	1	(3.3)
Middle-grade doctors	2	(8.0)	0	(0.0)	4	(13.3)
Assistant/Associate professor (university lecturer)	0	(0.0)	1	(4.0)	9	(30.0)
Professor	6	(24.0)	4	(16.0)	16	(53.3)
Discipline, *n* (%)						
General (family) medicine	3	(12.0)	3	(12.0)	2	(6.7)
Medical specialty	11	(44.0)	13	(52.0)	13	(43.3)
Surgical specialty	3	(12.0)	3	(12.0)	6	(20.0)
Anesthesiology-critical care	2	(8.0)	1	(4.0)	3	(10.0)
Paraclinical specialties	6	(24.0)	5	(20.0)	6	(20.0)
Experience (years), mean (SD)	11.57	(12.45)	7.40	(10.12)	21.88	(8.45)
Women, *n* (%)	13	(52.0)	17	(68.0)	16	(53.3)
**Standardized patients (** ***n*** **)**						
Status, *n* (%)						
Non-physician	10	(40.0)	15	(60.0)	11	(36.7)
Residents	6	(24.0)	0	(0.0)	0	(0.0)
Junior doctor	5	(20.0)	2	(8.0)	16	(53.3)
Middle-grade doctors	3	(12.0)	0	(0.0)	0	(0.0)
Assistant/Associate professor	1	(4.0)	3	(12.0)	3	(10.0)
Professor	0	(0.0)	5	(20.0)	0	(0.0)
Discipline, *n* (%)						
Not applicable	10	(40.0)	15	(60.0)	11	(36.7)
General (family) medicine	1	(4.0)	0	(0.0)	0	(0.0)
Medical specialty	5	(20.0)	6	(24.0)	7	(23.3)
Surgical specialty	3	(12.0)	2	(8.0)	7	(23.3)
Anesthesiology-critical care	4	(16.0)	1	(4.0)	4	(13.3)
Paraclinical specialties	2	(8.0)	1	(4.0)	1	(3.3)
Experience (years), mean (SD)	14.00	(NA)	21.00	(8.60)	2.67	(1.15)
Women, *n* (%)	13	(52.0)	11	(44.0)	13	(43.3)

### Distribution of the scores obtained by the students and association with their academic score

**Fig. 2:**
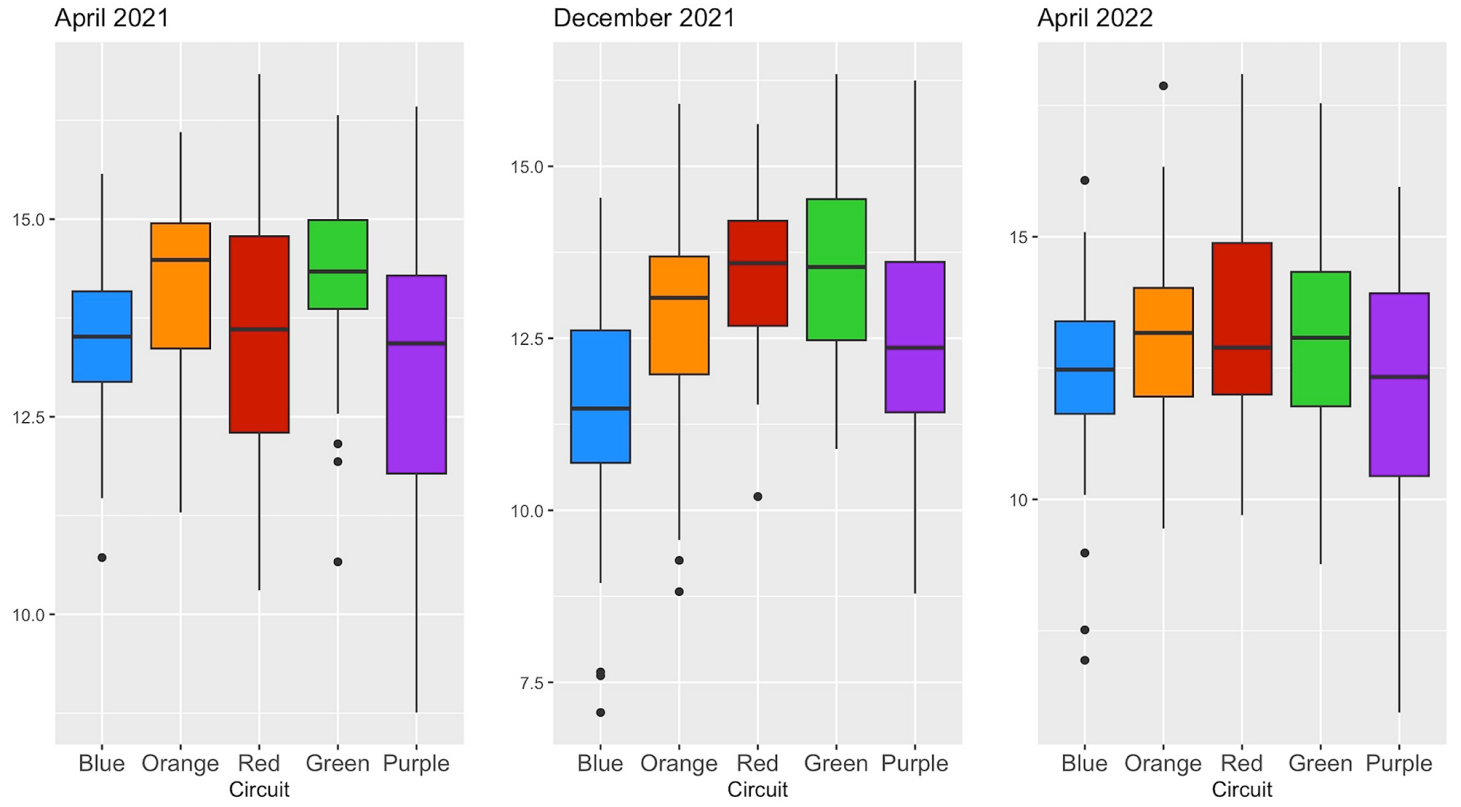
Distribution of the total score for each OSCEs session, per circuit

The mean ± SD total score obtained by the students on the OSCEs sessions in April 2021, December 2021, and April 2022 were respectively 13.7 ± 1.5, 12.7 ± 1.7 and 12.7 ± 1.9/20. The distribution of the total score was normal for each session. The distribution of the total score per session and per circuit is shown in Fig. 2.

Regarding the score obtained in a station, the largest difference between two circuits was observed for the station scenario relative to addiction medicine in December 2021, where the blue circuit obtained an average score of 9.6/20, while the students in the red circuit achieved an average of 14.7/20 (for an absolute difference of 5.1 points).

Table S1 (supplemental material) details the mean ± SD scores obtained on each station and in each circuit over the three sessions.

For the three sessions, there was a positive and significant linear relationship between the total score obtained by a student on the OSCEs session, and his/her average academic level (not including OSCEs). The beta coefficient (corresponding to the slope of the line) was + 0.49 in April 2021, + 0.64 in December 201 and + 0.61 in April 2022. Figure S1 (supplemental material) illustrates this relation per session and per circuit.

#### Multilevel analysis (variance due to the circuit)

The percentage of variance due to the circuit for April 2021, December 2021, and April 2022, was 4.5%, 22.3%, and 5.2% before adjustment, and 6.5%, 18.2%, and 3.8%, after adjustment for sex and the average academic level of students.

When comparing models including a random effect of the circuit (i.e., assuming a heterogeneity resulting from the examination conditions in addition to students’ performance) and those without, the likelihood ratio test was statistically significant for a random effect for December 2021 but not for the other sessions.

#### Factors associated with success in a station

The results of the multivariate analyses are given in Table 2. The score obtained by a given student on a given station was significantly related to the student’s average academic score, for all sessions. It was also significantly related to the clinical scenario (i.e., the topic being examined in a station).

**Table 2:** Multivariate analyses of the association between the score obtained by a student in a station and the characteristics of students, assessors, standardized patients, and the station’s clinical scenario

Variables	April 2021			December 2021			April 2022		
	ß Coefficient	SD	*p*-value**	ß Coefficient	SD	*p*-value**	ß Coefficient	SD	*p*-value**
Student’s mean academic score (excluding OSCEs) (coefficient variation for 1 point)	0.50	0.10	< 0.001	0.46	0.11	< 0.001	0.60	0.06	< 0.001
Student’s sex									
Man	0 (Ref)			0 (Ref.)			0 (Ref.)		
Woman	-0.21	0.25	0.39	0.43	0.25	0.09	-0.20	0.25	0.43
Station’s scenarios*			< 0.001			0.04			< 0.001
Station 1	0 (Ref.)			0 (Ref.)			0 (Ref.)		
Station 2	-0.71	0.50	0.16	2.30	1.53	0.17	1.53	0.94	0.12
Station 3	-3.27	0.52	< 0.001	3.55	1.46	0.04	-3.52	0.94	<0.01
Station 4	-2.06	0.51	< 0.001	0.14	1.81	0.94	-1.11	0.92	0.24
Station 5	-1.67	0.86	0.05	1.14	1.22	0.37	0.42	1.02	0.69
Assessors’s status			0.08			0.17			0.21
Junior doctor	0 (Ref.)			0 (Ref.)			0 (Ref.)		
Middle-grade doctor	0.41	0.48	0.40				2.33	1.67	0.18
Assistant/Associate professor (university lecturer)				1.36	1.62	0.42	1.68	1.67	0.33
University professor	-0.76	0.40	0.06	-1.44	0.90	0.14	3.05	1.68	0.09
Assessor’s specialty			0.02			0.31			0.95
General (family) medicine	0 (Ref.)			0 (Ref.)			0 (Ref.)		
Medical specialty	0.63	0.42	0.13	-0.70	0.96	0.48	0.00	1.09	1.00
Surgical specialty	0.94	0.55	0.09	-1.91	1.22	0.15	0.62	1.25	0.63
Anesthesiology-critical care	0.92	0.75	0.22	0.71	1.79	0.70	0.29	1.37	0.83
Paraclinical specialties	2.51	0.74	< 0.001	0.47	1.09	0.67	0.17	1.18	0.89
Assessor’s sex									
Man	0 (Ref.)			0 (Ref.)			0 (Ref.)		
Woman	-0.14	0.30	0.65	0.61	0.87	0.50	0.25	0.63	0.70
Status of the standardized patient									
Non-physician/resident/junior doctor	0 (Ref.)			0 (Ref.)			0 (Ref.)		
Senior physician	-2.74	0.49	< 0.001	0.27	1.40	0.85	-1.62	0.97	0.10
Sex of the standardized patient									
Man	0 (Ref.)			0 (Ref.)			0 (Ref.)		
Woman	-0.19	0.32	0.55	-2.46	1.24	0.08	-1.01	0.59	0.09

*A station number has been randomly assigned to the clinical situation assessed, it does not refer to an order of passage. Clinical situations and corresponding number are summarized in Fig. 1 for each session

** For categorical variables with more than two categories, the global *p*-value is shown in the variable name row. Other *p*-values refer to the comparison with the reference category

April 2021: random effects for “student” and “assessor/standardized patient pair” were non-significant (*p* = 0.19 and 0.79 respectively)

December 2021: random effects for “student” and “assessor/standardized patient pair” were statistically significant

April 2022: random effects for “student” and “assessor” were statistically significant. Random effect for standardized patient was non-significant (*p* = 0.20)

Regression coefficients are not available for assistant/associated professors in April 2021 and middle-grade doctors for December 2021 as these categories were not represented in the respective sessions

Furthermore, for April 2021, the scores obtained by the students were significantly better (by an average of + 2.51 points) when the assessor was a physician from a paraclinical specialty (with general medicine, i.e. family medicine, used as the reference). Also in the April 2021 session, the scores obtained by the students were significantly lower (by an average of -2.74 points) if the standardized patient was a senior physician (middle-grade doctor or higher), compared to a non-physician or physician with a junior profile (amateur actor, students with theatre experience, junior doctor). These two factors had no significant effect in the following two sessions. The sex of the students did not significantly affect the scores, although in December 2021, scores were numerically higher among female compared to male students (*p* = 0.09). The sex of the assessors was not related to the scores obtained. For the sessions in December 2021 and April 2022, the results appeared to be slightly worse, albeit without reaching statistical significance, when the standardized patient was a woman (*p* = 0.08 and 0.09 respectively).

Regarding the random effects for the score obtained in a station, the random effect related to the students was significant for December 2021 and April 2022. The random effect of participants in a station (assessor and standardized patient as a pair) was significant in December 2021 but not in April 2021. Finally, the distinguished random effect of the assessor and standardized patient (evaluated only in April 2022) was significant only for assessors.

## Discussion

This retrospective analysis of the first OSCEs sessions implemented at the Paris-Saclay medical school confirms a positive association between the scores obtained on the OSCEs and the student’s overall academic performance. The analysis showed that the highest percentage of the variance in the results of one session attributable to the examination condition was 18% in December 2021. In the first OSCEs session only, we observed a significant association between the scores obtained by the students in a station and both the assessor medical specialty and the standardized patients status. Our results confirm that while OSCEs are an excellent tool for assessing knowledge and skills among medical students, the objectivity and standardization of the scoring across students is never guaranteed, as previously reported by others [7–10].

Despite abundant literature discussing how best to judge the quality of OSCEs, the methodology used varies widely. Nevertheless, our results reporting the proportion of variance attributable to the examiners are in line with other studies that used similar methodology (multi-level analysis). In Canada, Sebok et al [11] estimated that 17% of the variance in results was attributable to the exam site location (and largely to the assessors) during OCSEs performed among international medical graduates seeking to qualify to practice in Canada via the Medical Council of Canada’s National Assessment Collaboration. In 2010, the Association for Medical Education in Europe (AMEE) published guidelines, including a literature review of tools used for measuring the quality of OSCEs [6]. The estimation of the random effect of the circuit, and thus, of the variance related to the assessors (potentially encompassing the site location and material used), is cited as a powerful metric for quality assessment, and an upper threshold of 30% of the circuit-related variance is proposed. In light of these recommendations, our results suggest that OSCEs implementation in our teaching hospital was properly conducted.

To ensure that the variance remains below this recommended threshold, it is essential to take several key measures to minimize the variance stemming from sources other than the students. Regarding the assessors, our findings show that the variance seems to stem from individual behaviors rather than from specific status, position or specialty. Indeed, many known biases in this type of examination could partially explain our results. Firstly, there is a variable degree of stringency (or conversely, leniency) in the grading between examiners, a phenomenon dubbed the “hawk-dove effect” [12]. We also cannot rule out the effect of any prior relations between the examiners and the students. Indeed, it has previously been shown that the scores are significantly higher when the examiner is familiar with the candidate [9]. While familiarity between assessors and candidates may be unavoidable within a single medical school, it should, in theory, be rendered moot by the participation of outside assessors (i.e. from outside the institution) during national-level exams. Our medical school is also working on creating a pool of standardized patients from the lay public, and thus, from outside our medical school, and theoretically unknown to the students. Finally, among the other known biases in OSCEs, the contrast effect is classically described, whereby a mediocre student is evaluated immediately after a series of candidates with lower level performance, thus resulting in an over-grading of the mediocre student purely by contrast with the previous, inferior candidates [10]. Certain assessors may fall prey to this phenomenon. The students’ overall level should thus probably be considered when scheduling the circuits to minimize this bias as much as possible.

According to the 5-category validity framework (content, response process, internal structure, relation with other variables and consequences) developed by Messicks [3], this study mostly explore two evidence sources of the validity of OSCEs. We studied the internal structure of our OSCEs sessions as our first objective was to assess how much of the students’ score was attributable to student performance alone, and we studied the relationship with other variables as we studied the association between student’s OSCEs score and mean academic score. Regarding the content of OSCEs, we did not measure it per se but it should be noted that the stations scenarios were produced and reviewed with a panel of experienced and specialized medical teacher. Finally, like most studies related to simulation-based assessment, our study did not examine the response process and the consequences, two of the five proofs of validity according to Messick’s framework [13]. As for Kane’s validity framework, the analyze of the first national OSCEs in France will be of great interest to confirm the choice of this examination method in the selection of the country’s future medical practitioners.

### Study limitations

One main limitation was the lack of data regarding certain aspects, due to the retrospective nature of the study. Indeed, a prospective design would have made it possible to verify and control the training and preparation of the examiners for the various sessions and stations and to include more explanatory variables in the models. Further analyses could focus on the type of competence expected on each station to determine whether the factors classically associated with success are identified in our population. For example, it has been shown that women obtain better scores on stations that assess communication and empathy [14].

Another limitation is related to the organization of these first sessions with fixed and unfixed pairs of participants (assessors and standardized patients) in a station, which prevented us from distinguishing between the assessors and standardized patient effects for two sessions. However, based on the total score obtained for each session, there doesn’t seem to be any difference between sessions with fixed and unfixed pairs.

Finally, our study may not have had sufficient statistical power to identify other variables significantly associated with student performance. For example, in the three sessions, we observed that the score obtained in a station was lower when the standardized patient was a woman. For December 2021 and April 2022, this observation was close to significance. An a posteriori simulation-based power calculation showed that our December 2021 dataset could only provide 70% statistical power to detect a score difference of − 2 when the standardized patients were women compared to men.

## Conclusion

The implementation of OSCEs requires rigorous methodology, in which the training and preparation of the examiners play a fundamental role in guaranteeing objective and efficacious scoring for the students. The variability in scores attributable to the examiners seems to be more related to individual behaviours in our study, rather than to any specific status, position or specialty. The examiner-related variance in our study was well below the threshold recommended by the AMEE. Standardization could be improved by creating a pool of standardized patients, and with the cumulating experience of the examiners in implementing this form of assessment, thereby contributing to reducing variance from sources other than student performance.

### Electronic supplementary material

Below is the link to the electronic supplementary material.

Supplementary Material 1

Supplementary Material 2

## Data Availability

The datasets used and/or analyzed during the current study are available from the corresponding author on reasonable request.
