# Supplementary material for: Variance due to the examination conditions and factors associated with success in objective structured clinical examinations (OSCEs): first experiences at Paris-Saclay medical school
Source: BMC Med Educ. 2024 Jul 2;24:716. doi: 10.1186/s12909-024-05688-5 (PMC11221172; doi:10.1186/s12909-024-05688-5)
Supplement: Supplementary file 1 — Supplementary Material 1 [file 12909_2024_5688_MOESM1_ESM.docx]

**Table S1.** Average score per station and per circuit during the three OSCE sessions

| **April 2021** | | | | **December 2021** | | | | **April 2022** | | | |
| --- | --- | --- | --- | --- | --- | --- | --- | --- | --- | --- | --- |
| **Station** | **Circuit** | **N** | **Mean±SD** | **Station** | **Circuit** | **N** | **Mean±SD** | **Station** | **Circuit** | **N** | **Mean±SD** |
| Cardio-respiratory arrest | Blue | 25 | 16.13 (1.33) | Addiction medicine | Blue | 36 | 9.62 (2.27) | Gastro-enterology | Blue | 42 | 12.93 (2.80) |
|  | Yellow | 25 | 14.85 (2.57) |  | Orange | 36 | 14.00 (2.72) |  | Orange | 41 | 13.86 (2.50) |
|  | Orange | 24 | 15.73 (1.37) |  | Red | 31 | 14.72 (2.24) |  | Red | 34 | 13.34 (3.05) |
|  | Red | 22 | 15.45 (2.23) |  | Green | 36 | 13.61 (2.41) |  | Green | 40 | 12.87 (3.31) |
|  | Green | 26 | 15.72 (2.29) |  | Purple | 36 | 11.14 (2.39) |  | Purple | 40 | 12.42 (2.76) |
| Cardiology | Blue | 25 | 13.26 (3.59) | Immunology | Blue | 36 | 11.21 (4.61) | Hypothyroidism | Blue | 42 | 14.94 (3.44) |
|  | Yellow | 25 | 11.16 (4.67) |  | Orange | 36 | 11.78 (3.66) |  | Orange | 41 | 13.90 (2.56) |
|  | Orange | 24 | 15.88 (3.84) |  | Red | 31 | 12.65 (3.58) |  | Red | 34 | 14.10 (2.11) |
|  | Red | 22 | 14.35 (3.90) |  | Green | 36 | 12.29 (3.34) |  | Green | 40 | 13.06 (3.02) |
|  | Green | 26 | 14.10 (3.36) |  | Purple | 36 | 12.58 (2.76) |  | Purple | 40 | 13.71 (3.25) |
| Pain | Blue | 25 | 12.16 (2.11) | Nephrology | Blue | 36 | 14.20 (2.84) | Infectious diseases | Blue | 42 | 10.45 (3.03) |
|  | Yellow | 25 | 13.63 (2.40) |  | Orange | 36 | 12.70 (2.49) |  | Orange | 41 | 11.17 (2.94) |
|  | Orange | 24 | 12.59 (1.88) |  | Red | 31 | 16.33 (1.90) |  | Red | 34 | 10.30 (4.60) |
|  | Red | 22 | 12.20 (2.38) |  | Green | 36 | 15.50 (1.87) |  | Green | 40 | 10.67 (3.38) |
|  | Green | 26 | 12.36 (2.20) |  | Purple | 36 | 14.01 (2.62) |  | Purple | 40 | 10.26 (4.13) |
| Fever | Blue | 25 | 12.38 (1.54) | Obstetrics | Blue | 36 | 8.46 (3.34) | Neurology | Blue | 42 | 10.77 (3.38) |
|  | Yellow | 25 | 12.26 (1.67) |  | Orange | 36 | 10.24 (3.16) |  | Orange | 41 | 11.61 (2.81) |
|  | Orange | 24 | 13.08 (1.37) |  | Red | 31 | 10.12 (2.11) |  | Red | 34 | 15.00 (2.73) |
|  | Red | 22 | 13.12 (1.65) |  | Green | 36 | 10.78 (2.82) |  | Green | 40 | 12.98 (3.89) |
|  | Green | 26 | 13.59 (2.21) |  | Purple | 36 | 11.11 (2.59) |  | Purple | 40 | 11.26 (2.81) |
| Imaging | Blue | 25 | 13.42 (2.64) | Rheumatology | Blue | 36 | 13.40 (2.49) | Respiratory medicine | Blue | 42 | 12.29 (3.77) |
|  | Yellow | 25 | 14.14 (3.64) |  | Orange | 36 | 15.25 (2.09) |  | Orange | 41 | 14.21 (3.06) |
|  | Orange | 24 | 13.65 (3.10) |  | Red | 31 | 13.26 (2.47) |  | Red | 34 | 14.18 (3.33) |
|  | Red | 22 | 12.72 (3.05) |  | Green | 36 | 15.55 (2.04) |  | Green | 40 | 15.16 (2.80) |
|  | Green | 26 | 14.90 (2.71) |  | Purple | 36 | 13.20 (2.43) |  | Purple | 40 | 12.70 (4.04) |

The lowest scores are highlighted in blue, and the highest scores are highlighted in red.
