# Supplementary figures and images for: Variance due to the examination conditions and factors associated with success in objective structured clinical examinations (OSCEs): first experiences at Paris-Saclay medical school
Source: BMC Med Educ. 2024 Jul 2;24:716. doi: 10.1186/s12909-024-05688-5 (PMC11221172; doi:10.1186/s12909-024-05688-5)

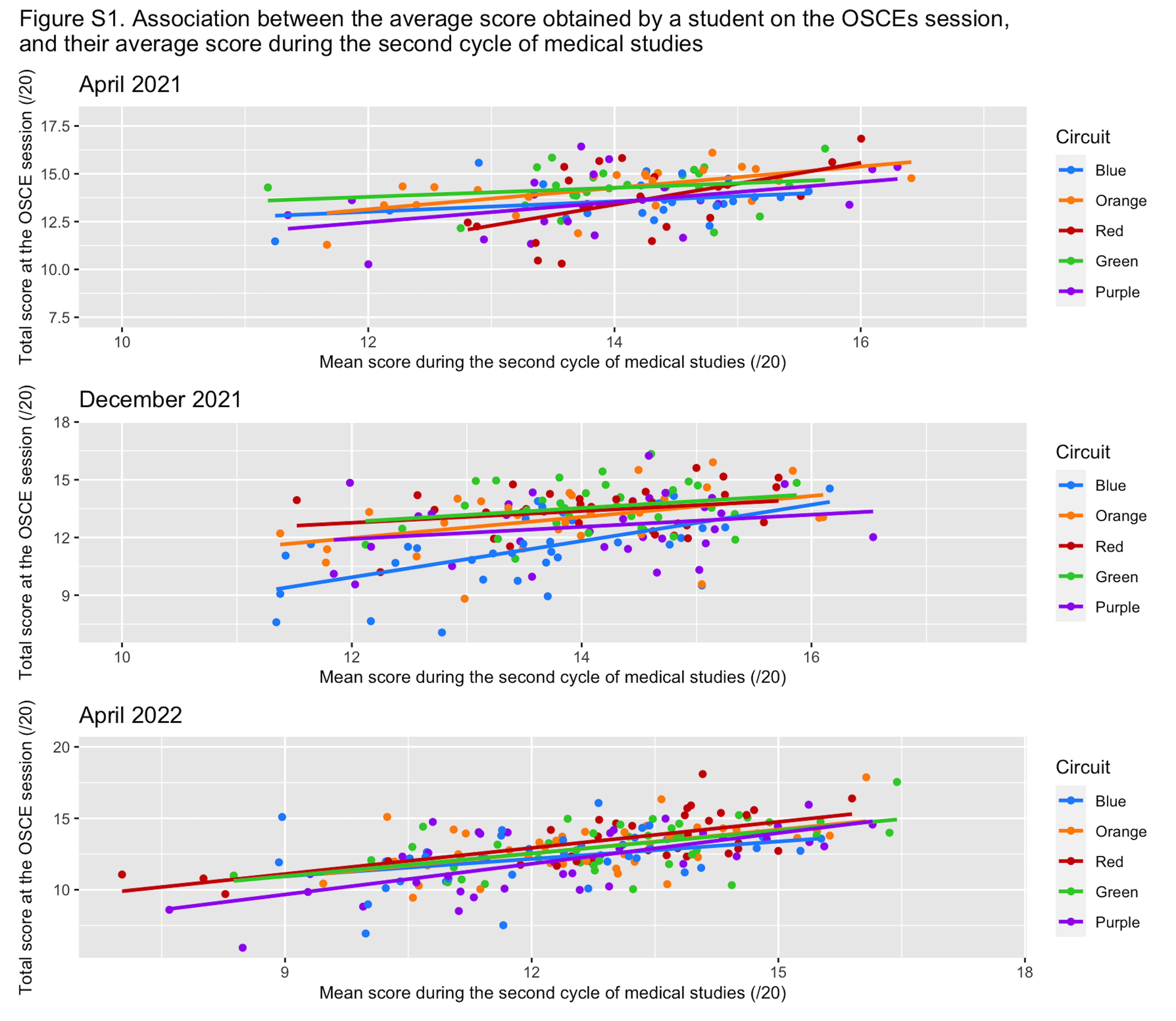

Supplement: Supplementary file 2 — Supplementary Material 2 [file 12909_2024_5688_MOESM2_ESM.jpg]
